# Pathological Features and Prognosis of Thymoma With or Without Myasthenia Gravis

**DOI:** 10.3389/fsurg.2022.726673

**Published:** 2022-02-18

**Authors:** Yunfeng Zhang, Lei Yu, Ji Ke

**Affiliations:** Department of Thoracic Surgery, Beijing Tongren Hospital, Capital Medical University, Beijing, China

**Keywords:** thymoma, myasthenia gravis, prognosis, surgical options, pathological features

## Abstract

**Introduction:**

To evaluate the different pathological and clinical characters of thymoma with and without myasthenia gravis (MG) and to determine whether the presence of MG influences the prognosis in patients with thymoma.

**Methods:**

Four hundred and twenty-five consecutive patients operated was analyzed. A median sternotomy was used in 189 cases, and video-assisted thoracoscopic thymectomy was used in 236 cases. These patients with thymoma were subdivided into two groups: thymoma with myasthenia gravis MG (*n* = 220) and thymoma without MG (*n* = 205). All thymic epithelial tumors were classified according to the WHO histologic classification and the Masaoka clinical staging system. The result was evaluated according to the Myasthenia Gravis Foundation of America's criterion. The clinical features of the 2 test were compared between the two groups, and the survival analysis of Cox treatment effects was compared between the two groups.

**Results:**

There were no perioperative deaths. The proportions of type A and thymic carcinoma were 0% in the group with MG and 10.7% (22/205) and 11.2% (23/205), respectively, in the group without MG. Thymic hyperplasia around the thymoma was 29.1% (64/220) in patients with MG and only 6.3% (13/205) in patients without MG (χ^2^ = 23.63, *P* = 0.000). The overall survival curve showed that the 5- and 10-year survival rates in the group without MG were 89.2 and 77.4%, respectively, while those in the MG group were 91.1 and 80.5%.

**Conclusions:**

The existence of MG has little influence on the prognosis of thymomas, but it is suitable for early diagnosis and treatment. Extended thymectomy should be performed on all patients with thymoma, whether they have MG or not.

## Introduction

Thymoma is often accompanied by paraneoplastic syndromes, such as hyperthyroidism, pure erythrocyte aplasia anemia, MG, and endocrinopathy. MG is the most common of these. Domestic and foreign data show that the proportion of MG combined with thymoma is 11.2–29.8%, while the proportion of thymoma combined with MG is 14.9–60.3%. There are differences in clinicopathological characters between thymoma combined with MG and thymoma alone. Academic circles have had disagreements about their outcome (1, 2). We retrospectively analyzed 425 patients with thymoma who underwent surgery in our hospital from January 2003 to December 2010 and discussed their clinical characteristics.

## Materials and Methods

### Study Subjects

From January 2003 to December 2010, 425 cases of thymoma were treated by thoracic surgery in Beijing Tongren Hospital, including 198 males and 227 females, aged 23 to 80, with a median age of 53. The course of disease ranged from 18 days to 4.5 years, with a median course of 9 months. Based on whether myasthenia gravis was combined, 220 patients were divided into a myasthenia gravis thymoma group (thymoma with MG group), and 205 patients were divided into a thymoma without MG group. The specific conditions of the two groups are shown in Table 1. Anti-acetylcholine receptor antibodies were positive in 141 patients (64.1%) in the MG group. The MG group was divided into 74 cases of type I, 43 cases of type IIa, 47 cases of type IIb, 24 cases of type IIIa, 25 cases of type IIIb, four cases of type IVa and three cases of type IVb according to the clinical classification of the American Myasthenia Gravis Foundation of America (MGFA) (3, 4). In the pre-operative MG group, 185 patients (84.1%) were treated with bromo-pyridostigmine, and 89 patients (40.5%) were treated with glucocorticoids.

**Table 1:** Comparison of clinicopathological features between thymoma patients with and without myasthenia gravis.

**Group**	**Number of cases**	**Male/female**	**Age**			**Masaoka clinical stage (number)**				**Sternotomy**	**Thoracoscopic surgery**
			**>60**	**41**~**60**	**≤40**	**I**	**II**	**III**	**IV**		
Thymoma with MG	220	98/122	56	126	38	74	90	49	7	99	121
Thymoma without MG	205	100/105	48	133	24	58	76	55	16	112	93
Chi-square value	-	1.289	1.522	1.494	1.427	2.133	1.663	1.473	1.637	1.433	1.622
*P-*value	-	0.621	0.389	0.474	0.376	0.372	0.528	0.445	0.531	0.421	0.489
**Group**	**Combined hyperthyroidism**	**WHO Pathological Classification**						**Histopathology of thymus around tumor**			**Unresected cases**
		**TypeA**	**AB**	**B1**	**B2**	**B3**	**Thymic carcinoma**	**Thymic atrophy**	**Thymic hyperplasia**	**microscopic thymoma**	
Thymoma with MG	52	0	49	58	67	46	0	137	64	7	12
Thymoma without MG	9	22	31	43	53	33	23	171	13	1	20
Chi-square value	12.310	16.225	3.551	2.405	1.956	1.623	18.142	—	23.630	—	4.325
*P-*value	0.000	0.000	0.059	0.120	0.161	0.202	0.000	—	0.000	—	0.028

### Research Methods

**Figure 1:**
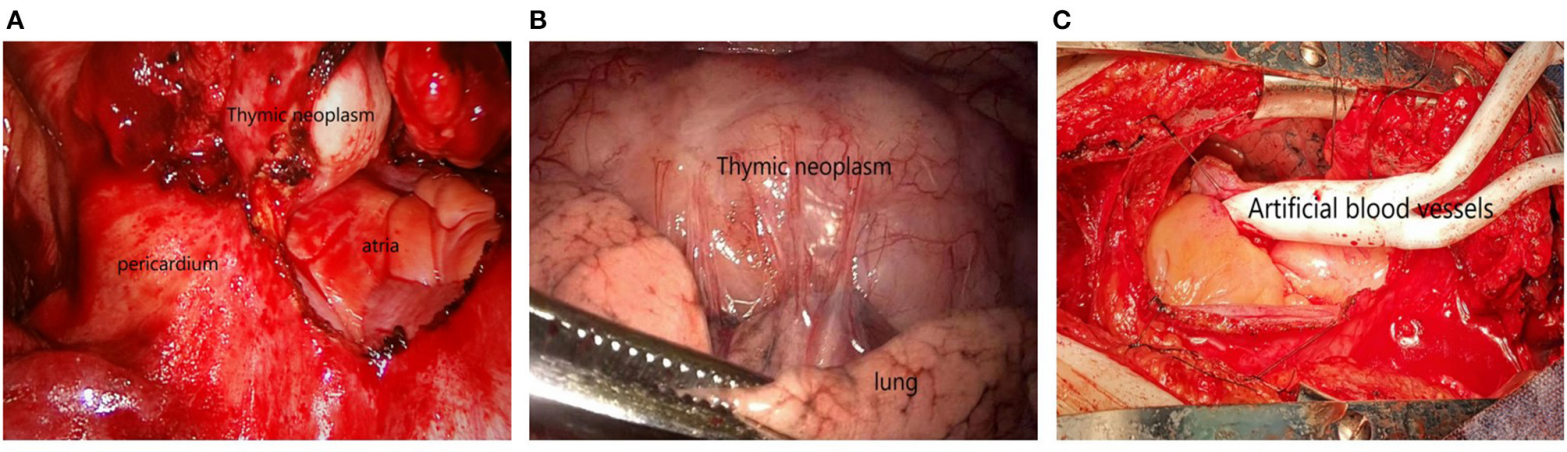
**(A)** Tumor invasion of pericardium; **(B)** Tumor invasion of lung tissue; **(C)** The tumor invaded the innominate and superior vena cava, and the tumor was resected followed by artificial vascular replacement.

Surgical approach: 189 patients underwent median sternotomy (5). Double-lumen endotracheal intubation was used for the operation. The patient was placed in the supine position, and a median incision was made from the upper sternal notch to the xiphoid process. The sternum was split longitudinally from bottom to top along the median line with an electric saw, and distracted by a sternal retractor to expose the mediastinal area. The bilateral phrenic nerves were clearly distinguished, the mediastinal pleura was opened, and the right inferior pole of the thymus was freed upward to the confluence of the right internal mammary vein into the superior vena cava. The right upper pole was completely removed at the angle between the superior vena cava and the intramammary vessels. The innominate vein was carefully dissected, the thymus nourishing vessel was found, and the proximal end was clipped with a titanium clip and cut off with a supersonic knife. The left lobe of the thymus was treated with the same method. The thymus and thymic tumors were completely excised, and the fat of each group of the anterior mediastinum was removed. Partial pericardiectomy or pulmonary wedge resection is necessary for tumors invading pericardium and lung tissue (see Figure 1).

Another 236 patients underwent enlarged thymectomy and thymoma resection by thoracoscopy (6). All patients in the thoracoscopic surgery group underwent a unilateral thoracic approach. General anesthesia/double-lumen intubation was used for the operation. The left or right approach was decided according to the location of the thymic tumor. In the right approach, the patient was placed in the left lateral decubitus position. Under the guidance of thoracoscopy after lung collapse, 1.5 cm incisions were made at the 2nd intercostal space and the 4th intercostal space of the ipsilateral axillary anterior line, respectively, as the operation hole. The mediastinal pleura was opened and the thymus was completely free from the sternal surface. The right upper pole was completely removed at the angle between the superior vena cava and internal mammary vessels. The innominate vein was carefully dissected, the thymus nourishing vessel was found, and the proximal end was clipped with a titanium clip and cut off with a supersonic knife. The left lobe of the thymus was treated in the same way. The thymus was completely excised, and the fat in each group of the anterior mediastinum was removed. Fat removal at the base of the neck was performed through a 2–3 cm transverse incision at the neck to remove fat from the pretracheal fascia (see Table 1 for details). Extended thymectomy refers to the removal of adipose tissue and ectopic thymus in the cervical root and anterior mediastinum groups in addition to complete thymectomy (3). Unresected cases included partial tumor resection and simple biopsy.

Tumor classification, staging and treatment: All thymic tumors were classified and staged according to WHO (2004 Edition) tissue classification (7) and modified Masaoka clinical stage (8). Type A and stage I thymoma need no adjuvant treatment except an operation. Patients with type AB, B1, or stage II and III thymomas were treated with mediastinal radiotherapy. Radiotherapy and chemotherapy were started 1 month after operations for stage IV thymoma and thymic cancer. The curative effect for patients with MG was evaluated according to MGFA standards (4).

### Follow-Up Method

Follow-up was completed by telephone, letter, email, and outpatient service. The follow-up time was 3, 6 months, 1, 2, 3, 5, and 10 years after the operation. The follow-up period was 7–12 years, with an average of 9.5 years. The follow-up included checking current symptoms, medication, imaging data, and blood examination.

### Statistical Methods

SPSS 19.0 software was used to analyze the data. The measurement data of normal distribution was expressed with, and the comparison between groups was conducted by *t*-tests. The measurement data of non-normal distribution was expressed by M (QR), and the comparison between groups was conducted by rank–sum test. The count data were expressed by frequency and percentage, and the comparison between groups was conducted by Chi-square tests. The survival curve was compared by Cox survival analysis. The difference was statistically significant (*P* < 0.05).

## Results

### Surgical Results

There was no intraoperative death in any patients. Among 32 patients with unresectable thymoma (12 with MG, 20 without MG), 11 patients with thymoma invading superior vena cava or pulmonary vessels, and 21 patients with pleural and pericardial metastasis. In 393 cases, the thymus and tumor were completely removed. There were 47 cases (12.0%) of thymoma invaded pericardium, 35 cases (8.9%) of lung adhesion, and 29 cases (7.4%) of left innominate vein invaded. There was no difference in patients' ICU and hospital stays between the two groups. In 75 cases (19.1%), patients had post-operative complications, including 43 patients with MG who experienced myasthenic crisis, and 19 cases of pneumonia, six cases of pulmonary embolism, and seven cases of sternal incision infection (see Table 2).

**Table 2:** Surgical results and complications data.

	**Thymoma with MG (*n* = 220)**	**Thymoma without MG (*n* = 205)**	***P-*value**
Unresectable thymoma	12	20	0.028
Operation time (min)	112.35 ± 20.46	115.29 ± 23.75	0.171
Intensive care unit length of stay (d)	6 ± 1.5	7 ± 2.5	6.943
hospital length of stay (d)	9 ± 2.5	11 ± 3.0	4.188
**Complications**			
Myasthenic crisis	36	7	0.000
Pneumonia	11	8	0.584
Pulmonary embolism	3	3	0.931
Sternal incision infection	4	3	0.774

### Pathological Examination Results

The proportion of type A thymoma and thymic carcinoma in the combined group was 0%, while that in the thymoma without MG group was 10.7 and 11.2%, respectively. The results of pathological examination of thymus tissue around the tumor showed that the proportion of thymus hyperplasia was 29.1% (64/220) in the MG group and 6.3% (13/205) in the group without MG (χ^2^ = 23.63, *P* = 0.000). In the MG group, a small thymoma was found in seven cases around the tumor, with a maximum diameter of 2–5 mm.

Masaoka clinical staging results: in the MG group, stage III and IV thymoma accounted for 25.5% (56/220). In the group without MG, stage III and IV accounted for 34.6% (71/205) (χ^2^ = 1.785, *P* = 0.163).

### Follow-Up Results

There were 50 patients lost to follow-up. Among 375 patients who were followed up, 208 cases were in the MG group and 167 cases in the group without MG. The overall survival curve showed that the 5- and 10-year survival rates in the group without MG were 89.2 and 77.4%, respectively, while those in the MG group were 91.1 and 80.5%. Eight patients died of non-thymoma related diseases (four of heart disease, two of kidney disease, and two of cerebral vascular disease). Within 5 years of the operation, 17 patients in the MG group died. Of these, 10 died of myasthenic crisis (three patients died of myasthenic crisis during radiotherapy, and seven patients died of recurrence and metastasis of thymoma). In the group without MG, 12 patients died. Of these, nine died from stage III or IV thymoma or thymic carcinoma.

In the MG group, the complete remission (CR) rate of myasthenia gravis was 21.6% (45/208) in the second year after the operation. The CR was increased to 31.3% (65/208) in the fifth year after the operation. In the second year after the operation, the effective rate was 75.9% (158/208), with no change in MG in 41 cases and aggravation in nine cases.

### Correlation Between Histological Type, Clinical Stage, and Prognosis

No recurrence was found in patients with type A thymoma. Among the 183 cases with complete resection, 22 cases (10.5%) relapsed in the MG group and 20 cases (12.1%) relapsed in the group without MG. There was no significant difference between the two groups (χ^2^ = 0.256, *P* = 0.637). According to the analysis of thymomas in the group without MG, the proportion of stage III and IV increased with the increase of the malignant degree of thymic tumors, which was 33.96% for type B2, 57.58% for type B3 and 100% for type C (see Table 3). Tumor stage directly determines the survival and prognosis of patients. The 5- and 10-year survival rates of stage III and IV thymoma patients without MG were 86.23 and 68.41%, respectively, which were significantly lower than 98.56 and 89.48% of stage I and II patients (*P* = 0.023).

**Table 3:** Analysis of histological and clinical types of thymomas in patients without MG.

**Without MG (205)**	**A (22)**	**AB (31)**	**B1 (43)**	**B2 (53)**	**B3 (33)**	**C (23)**
I (58)	16 (72.73%)	15 (48.39%)	13 (30.23%)	8 (15.09%)	6 (18.18%)	0
II (76)	6 (27.27%)	14 (45.16%)	21 (48.84%)	27 (50.94%)	8 (24.24%)	0
III (55)	0	2 (6.45%)	7 (16.28%)	13 (24.53%)	16 (48.48%)	17 (73.91%)
IV (16)	0	0	2 (4.65%)	5 (9.43%)	3 (9.09%)	6 (26.09%)
				13 + 5 (33.96%)	16 + 3(57.58%)	100%

**Figure 2:**
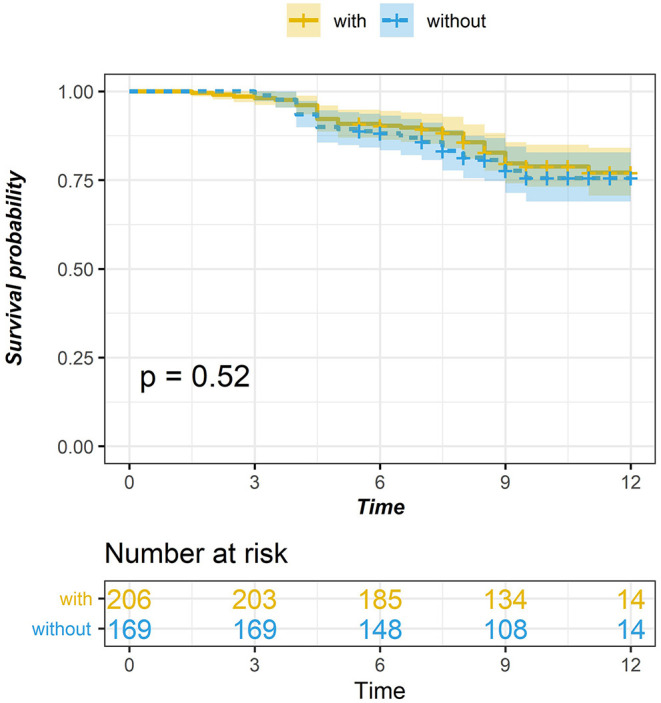
Comparison of survival curve between thymic tumor and thymoma with myasthenia gravis (*P* = 0.784).

Through the study of the survival curves of the two groups, it is found that although the follow-up data after 5 years show that the survival time of thymoma patients without MG is slightly lower than that of thymoma patients in MG group, there is no significant difference in the long-term survival rate between the two groups, and the existence of myasthenia has no significant impact on the prognosis of thymoma patients (see Figure 2).

## Discussion

The impact of MG on the prognosis of thymoma has been the focus of debate in academia for some time. Academia has considered MG as an unfavorable prognostic factor of thymoma, which increased perioperative and post-operative mortality by inducing myasthenic crisis, and other issues. Moreover, the tumor classification has a certain impact on the occurrence of perioperative myasthenic crisis. The incidence of perioperative myasthenic crisis in type A thymoma is lower, while the incidence of type AB and B is higher (9–11). However, in recent years, some scholars believe that the occurrence of MG is conducive to early diagnosis, a high success rate of surgical resection, and a good prognosis (2, 3, 9). We retrospectively analyzed 425 thymoma patients who underwent surgery in our hospital from January 2003 to December 2010, and this should be one of the longest follow ups in the literature. The results of our study showed that the 5-year survival rate of the MG group was slightly lower than that of the group without MG, and the 10-year survival rate and tumor recurrence rate were similar. In the MG group, 10 cases died due to myasthenia crisis, which were the aggravation of new myasthenia or the progression of original myasthenia symptoms, most of which were caused by tumor recurrence. From this, it can be seen that the long-term prognosis of thymoma patients with myasthenia was poor and the mortality was relatively high. After analysis, it was believed that the patients with myasthenia gravis were diagnosed in our hospital mostly because of the ptosis of the upper eyelids, while the patients in the group without MG were mostly treated after the occurrence of local compression or invasive symptoms (such as chest pain, cough, and even dyspnea). The patients in stage III and IV of the thymoma group without MG accounted for 34.6%, which was higher than that in the group with MG (25.5%). The number of unresected cases was also higher. We believe that thymoma combined with MG has little effect on its long-term efficacy. However, for some patients with symptoms of MG first diagnosed with thymic tumors, the tumors are at a relatively early stage, greatly improving the resection rate of tumors. The prognosis is also ideal.

In addition to the complete resection of thymic tumors, we advocate enlarged thymectomy, i.e., complete resection of thymus and removal of fat and ectopic thymus in the cervical root and anterior mediastinum groups, whether through a median sternotomy or thoracoscopic surgery (11). We believe that myasthenia gravis is not entirely thymoma-induced, and 29.1% of patients in the MG group had hyperplasia of thymic tissue around the thymoma, compared with only 6.3% in the group without MG. In some patients, the proliferation of thymic tissue around the tumor may be the real cause of MG. In addition to the presence of microthymoma in the ectopic thymus (12), thymoma resection alone or thymectomy is not conducive to the treatment of MG. In some cases, new MG occurs post-operatively (13) and may even induce myasthenic crisis post-operatively. Therefore, whether combined with MG or not, enlarged thymectomy should be the standard surgical approach for thymoma treatment.

In addition, complete thymectomy is important for long-term outcomes in patients with thymoma (14–16). Nine of the 12 deaths in the group without MG 5 years after surgery died of unresected stage IV thymoma or thymic adenocarcinoma.

When analyzing the post-operative pathological data, we also found no type A thymoma and thymic adenocarcinoma in the MG group. Domestic and foreign reports show that the incidence of type A thymoma is 0–14%, type AB is 6–42%, type B1 is 7–50%, type B2 is 24–71%, and type B3 is 25–65%, and there is almost no MG in patients with thymic cancer (8, 17–21). In this study, the proportion of type A thymoma and thymic adenocarcinoma in the group without MG was 10.7 and 11.2%, respectively, and the proportions of type B1 and B2 thymoma in the two groups were basically similar.

The combination of MG has little effect on the long-term efficacy of thymoma patients, but the presence of MG is conducive to the early diagnosis and treatment of thymoma. Surgery for thymoma with or without MG should be performed with thymoma and enlarged thymectomy. Type A thymoma and thymic adenocarcinoma are rarely associated with MG. Myasthenic crisis, stage IV thymoma, and thymic adenocarcinoma were the main causes of death in the thymoma group with MG and thymoma alone, respectively.

## Data Availability Statement

The original contributions presented in the study are included in the article/supplementary material, further inquiries can be directed to the corresponding author.

## Ethics Statement

The studies involving human participants were reviewed and approved by the Ethics Committee of Beijing Tongren Hospital. The patients/participants provided their written informed consent to participate in this study.

## Author Contributions

YZ conceived of the study. LY participated in its design and coordination. JK helped to draft the manuscript. All authors read and approved the final manuscript.

## Conflict of Interest

The authors declare that the research was conducted in the absence of any commercial or financial relationships that could be construed as a potential conflict of interest.

## Publisher's Note

All claims expressed in this article are solely those of the authors and do not necessarily represent those of their affiliated organizations, or those of the publisher, the editors and the reviewers. Any product that may be evaluated in this article, or claim that may be made by its manufacturer, is not guaranteed or endorsed by the publisher.
